# A Comparison Between the Residual Stresses of Ti6Al4V and Ti-6Al-2Sn-4Zr-6Mo Processed by Laser Powder Bed Fusion

**DOI:** 10.3390/ma18030689

**Published:** 2025-02-05

**Authors:** Alberta Aversa, Alessandro Carrozza, Vincenza Mercurio, Flaviana Calignano, Olha Sereda, Vaclav Pejchal, Mariangela Lombardi

**Affiliations:** 1 Department of Applied Science and Technology, Politecnico di Torino, Corso Duca Degli Abruzzi 24, 10129 Torino, Italy; alessandro.carrozza@polito.it (A.C.); mariangela.lombardi@polito.it (M.L.); 2 Consorzio Interuniversitario Nazionale per la Scienza e Tecnologia dei Materiali (INSTM), Via G. Giusti 9, 50121 Firenze, Italy; 3 Department of Management and Production Engineering (DIGEP), Politecnico di Torino, Corso Duca Degli Abruzzi 24, 10129 Torino, Italy; vincenza.mercurio@polito.it (V.M.); flaviana.calignano@polito.it (F.C.); 4 Centre Suisse d’Electronique et de Microtechnique (CSEM), Jaquet Droz 1, CH-2002 Neuchȃtel, Switzerlandvaclav.pejchal@csem.ch (V.P.)

**Keywords:** additive manufacturing, titanium alloys, Ti6246, Ti6Al4V, residual stress

## Abstract

Metal additive manufacturing processes induce residual stress in as-built components. These residual stresses are detrimental to part quality as they can induce defects such as warping and delamination. In some cases, when complex components are built, residual stress can even cause a build job to fail due to the recoater crashing into the distorted part. In this paper, the residual stress values of Ti6Al4V and Ti-6Al-2Sn-4Zr-6Mo alloys were evaluated by the cantilever approach and by the X-ray diffraction sin^2^(Ψ) method. The results showed that, as expected, Ti6Al4V as-built cantilevers displayed high distortion and von Mises equivalent stress values up to 494 MPa. On the contrary, as-built Ti-6Al-2Sn-4Zr-6Mo cantilevers were characterized by almost null warping and a residual stress value in the as-built state of 191 MPa. This different behavior is mainly due to the different properties of the hexagonal α’ martensite in Ti6Al4V and the soft orthorhombic α’’ martensite in Ti6246. The post-processing heat treatment significantly reduced the residual stress in Ti6Al4V, lowering it to 44 MPa, while, in the case of Ti-6Al-2Sn-4Zr-6Mo, the post-processing heat treatment did not affect the residual stress conditions. These findings suggest that Ti-6Al-2Sn-4Zr-6Mo could be a suitable candidate for the additive manufacturing production of extremely complex parts, as it could reduce the risks associated with recoater crashes and job failures.

## 1. Introduction

Residual stresses in metal additive manufacturing (AM) components strongly influence part quality in terms of geometrical accuracy and material properties. On the one hand, residual stress may in fact cause distortions and lead to build job interruptions, delamination from the support structures, and thin-wall warping. These effects limit the geometrical freedom inherent to the AM processes and force designers to simplify the component drawings [1]. On the other hand, residual stress also strongly alters the properties of as-built alloys as it generally lowers their chemical resistance, magnetization, and static and dynamic strength [2]. The reduction in these properties reduces the probability of AM part acceptance as they might not meet the end users’ requirements.

Residual stresses are due to the complex thermal history of AM processes and in particular due to the layer-by-layer aspect and to the rapid heating and cooling steps. Two mechanisms have been proposed for the formation of these stresses: the temperature gradient mechanism and the cool-down phase. Based on the first mechanism, due to laser scanning, a steep thermal gradient is generated, and the material strength is reduced where high temperatures are observed. During heating, the expansion of the top layer is restricted by the underlying material, generating compressive stresses. When these stresses reach the yield strength of the material, plastic deformation will arise. During cooling, this material will shrink, causing counter-bending and tensile stress in the top layer and compressive stress in the lower part [3]. According to the cool-down phase mechanisms, after the laser scanning, the last layer shrinks due to cooling and this shrinkage is hindered by the previous layers. This causes the formation of tensile stress in the top layers and compressive stresses in the lower ones [2]. Based on these mechanisms, it is evident that residual stresses in AM parts are strongly related to the thermo-mechanical properties of the material, such as thermal conductivity, phase transition temperatures, Young’s modulus, and yield strength. These properties determine, indeed, the temperature gradient, solidification and cooling shrinkage, the intensity of the induced stresses, and the ability to withstand them without deformation. Among the different alloys, titanium alloys show the highest residual stress values due to their low thermal conductivity and consequent high thermal gradient, Young’s modulus, and yield strength [4,5]. Furthermore, the effect of the building conditions on the residual stresses of titanium alloys is quite complex due to the changes in volume and properties related to the allotropic transformation from β to martensite during cooling [6].

Titanium alloys, however, find applications in various fields such as the aerospace, automotive, and biomedical industries. The main applications of these alloys match well with the intrinsic advantages of AM related to complex shapes and customized parts. Because of these reasons, this alloy class is among the most used in the AM field. Ti6Al4V is by far the most used titanium alloy and its success is mainly due to its combination of physical and mechanical properties [7]. Ti-6Al-2Sn-4Zr-6Mo, also known as Ti6246, has also been recently introduced in the AM market and is seeing substantial success thanks to its high strength and easy AM processability. Up to now, only a few papers have been published on this material processed by AM [8,9,10,11,12]. At first, Carrozza et al. showed that this alloy possesses excellent processability by Laser Powder Bed Fusion (LPBF) and that low-porosity samples can be built using a wide range of process parameters [9]. Furthermore, Ti6246 alloy appears to be suitable for the production of thin features [12]. Thanks to the presence of β-stabilizing elements, such as Mo, Nb, and Ta, in the as-built state, this alloy is constituted by soft orthorhombic martensite (α″). Carrozza et al. showed that Ti6246 properties can be easily tailored using post-processing heat treatments by tuning the martensite decomposition [8]. Pirro et al. also focused on post-processing heat treatments and optimized the solution treatment and ageing of Ti6246 processed via LPBF [13]. Peng et al. also demonstrated that this alloy possesses an extremely rapid hardening response and that ultra-high hardness values can be achieved thanks to the decomposition of martensite in fine α + β [11].

This paper aims to evaluate the residual stresses of a Ti6246 alloy and compare them with those of Ti6Al4V built in conventional process conditions. The cantilever method was used as it is quite straightforward and indicates type I stress. The sin^2^(*Ψ*) X-ray diffraction (XRD) method was also used to validate and quantify the cantilever results. The XRD method is localized and evaluates type II residual stress.

## 2. Materials and Methods

Ti6Al4V and Ti-6Al-2Sn-4Zr-6Mo samples were produced using powders provided by EOS (EOS GmbH, Krailling, Germany) and TLS (TLS Technik GmbH & Co. Spezialpulver KG Bitterfeld-Wolfen, Germany), respectively. The chemical compositions of the powders are reported in Table 1. All the samples were produced using an EOS M270 extended system (EOS GmbH, Krailling, Germany) using the building parameters optimized in previous works and reported in Table 2 [9]. The building parameters were optimized based on the densification of samples and it is important to underline that they were characterized by different Volumetric Energy Densities (VEDs). Some samples underwent post-processing heat treatments in a VF800/S high-vacuum furnace (Pro.Ba. Srl, Cambiano, Italy). The details of these heat treatments, which were selected as they are the most commonly used ones for these alloys and are both below the β-transus temperatures, are reported in Table 3. The 50 °C difference among these heat treatments is in line with the difference among their β-transus temperatures [14]. The duration of the heat treatment was selected as 2 h as it is suggested by the Ti6Al4V powder provider and it is the most commonly used one in the literature [7].

**Figure 1:**
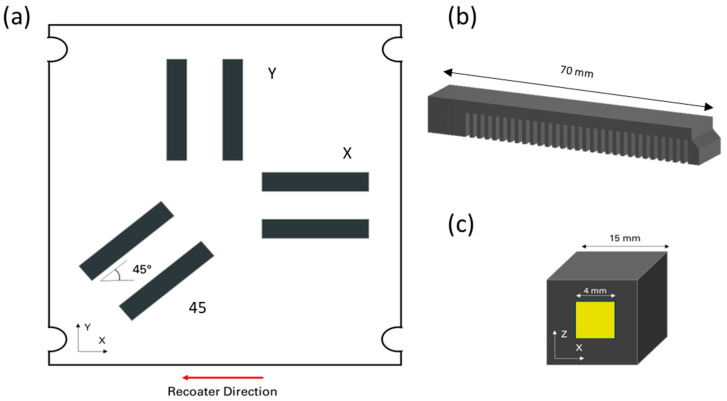
Schematic representation of the (**a**) cantilever samples on the building platform, (**b**) a cantilever sample, and (**c**) a cube in which the yellow area indicates the region where the XRD measurement was performed.

In the first job, 15 × 15 × 15 mm^3^ cubes were built to evaluate the alloys’ microstructures. Subsequently, two jobs with cantilever samples with different orientations (X, Y, and 45°) were performed using each material (Figure 1). One of these platforms per alloy underwent the heat treatments reported in Table 3. The as-built (AB) samples were measured using a DEA IOTA coordinate-measuring machine (Hexagon AB, Stockholm, Sweden).

Cantilever samples were then cut using wire electrical discharge machining (WEDM), using a DK7732 system by Suzhou Baoma Numerical Control Equipment (Suzhou, China), using a current (that is the number of power tubes) value of 3, a pulse off-time of 7 µs, and a pulse on-time of 12 µs.

The cubic samples were cut along the building direction, ground, and polished using colloidal SiO_2_ suspension, water, and H_2_O_2_. Microstructural characterizations were conducted after etching the polished cross-sections with Kroll solution (93% H_2_O, 5% HNO_3_, 2% HF). The specimens were observed by means of an optical microscope (OM) by Leica DMI 5000 M OM and a scanning electron microscope (SEM) by Phenom-XL (EDS, Phenom XL, Thermo Fisher Scientific, Waltham, MA, USA). Electron backscatter diffraction measurements were carried out by using a TESCAN 2900G FIB-SEM microscope (Tescan, Brno, Czech Republic), using 40 keV and 10 nA and a step size of 0.5 μm.

XRD measurements were performed using a Panalytical X’Pert Materials Research Diffractometer (Malvern Panalytical, Almelo, the Netherlands) equipped with a parallel-beam mirror and parallel-plate collimator with Cu Kα radiation in the middle of the polished XZ planes within an approximately 4 × 4 mm^2^ area, as illustrated in Figure 1c. With this radiation, the penetration depth is approximately 20 μm. Prior to the residual stress measurements, 2θ/ω scans were performed for the phase analysis of each investigated material and heat treatment condition.

For the residual stress measurement, the sin^2^*ψ* method was performed. This involves measuring the diffraction angles of a selected reflection at various tilt angles (*ψ*). Selecting strong reflections at higher 2θ angles, ideally above 90°, is recommended to achieve higher precision. The primary assumptions of this method include the plain stress condition, where stress is considered to be uniform across the analyzed surface area and to be zero normal on the surface of the specimen. Since the XRD deals with the near-surface residual stress, this assumption is reasonable. Thus, the stress distribution is described by the principal stresses *σ*_1_ and *σ*_2_ in the plane of the surface with the stress perpendicular to the surface *σ*_3_ = 0. However, a strain component perpendicular to the surface, ε_3_, has a non-zero value as a result of the Poisson ratio contractions caused by the biaxial stress state along the surface. To quantify the strain and, consequently, the stress along a given rotation angle φ, the interplanar spacing between crystallographic planes is measured at several tilt angles (*ψ*).

To quantify the full biaxial stress distribution, measurements of the stress along at least three angles φ are necessary. For simplicity, residual stress along *φ* for an isotropic linear elastic material with Young’s modulus E and Poisson’s ratio *ν*, assuming a biaxial stress state, can be calculated as [15]

$$\frac{d_{\varphi \psi }-d_{0}}{d_{0}}=\frac{1}{2}s_{2}\sigma_{\varphi }\sin^{2}\psi +\frac{1}{2}s_{2}\sigma_{13}\sin2\psi +s_{1}\left(\sigma_{11}+\sigma_{22}\right)$$

in which *d*_*φψ*_ is the interplanar spacing for *φ* direction and *ψ* tilt, the constants $s_{1}=-\frac{\nu }{E}$ and ${\frac{1}{2}s}_{2}=\frac{1+\nu }{E}$ are the X-ray elastic constants (XECs) and *d*_0_ is the interplanar spacing for the stress-free material. This equation indicates a linear correlation between *d*_*ψ*_ and sin²*ψ* in the absence of surface shear stress (σ_13_ = 0). Consequently, *σ_φ_* can be determined from the slope of the *d*_*ψ*_ versus sin²*ψ* plot using linear regression. Due to the difficulty in determining the lattice spacing of a stress-free material, the lattice spacing at the first tilt angle is used as *d*_0_, which results in an approximate 2% error in the residual stress calculation [16]. 

For the α or α’ Ti phase, the (211) reflection near 2θ = 110° was selected for residual stress analyses. For orthorhombic α″, as a dominant phase present in as-built Ti6246, the (221) reflection near 2θ = 77° was used to determine the residual stress. A scan range of 8° around the selected peak positions for residual stress analysis with a 0.075° step size was performed. The position of the selected peaks was measured in four different rotation angles *φ* with respect to the sample reference frame, that is 0°, 45°, 90° and 135°. For each direction, measurements at 10 different tilt angles *ψ* were performed covering the range sin^2^(*Ψ*) from 0 to 0.9. To calculate residual strain, the measured lattice strain diffraction elastic constants (XECs) *s*_1_ = 3.22 TPa^−1^ and $\frac{1}{2}$*s*_2_ = 12.75 TPa^−1^ corresponding to the (211) reflection of hexagonal-α′ were considered [17]. In the case of Ti6246 in the as-built condition, due to lack of values in the literature for the XEC of the orthorhombic α″, an isotropic behavior was assumed with E = 115 GPa and *ν* = 0.32.

## 3. Results and Discussion

**Figure 2:**
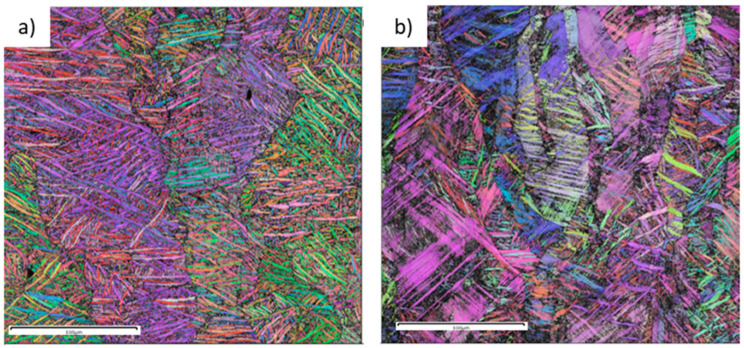
EBSD IPF maps of (**a**) Ti6Al4V and (**b**) Ti6246 as-built samples along the XZ plane.

**Figure 3:**
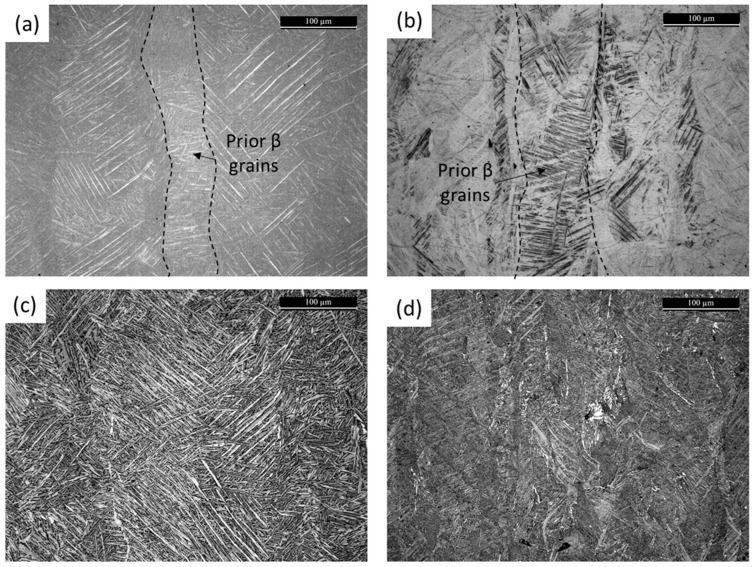
Micrographs of (**a**) Ti6Al4V and (**b**) Ti6246 as-built and (**c**) Ti6Al4V and (**d**) Ti6246 heat-treated samples along the XZ plane.

The microstructural features of Ti6Al4V and Ti6246 in the as-built (AB) state were analyzed via EBSD and OM microscopy (Figure 2 and Figure 3a,b). In both cases in the AB state, elongated prior β grains can be detected. EBSD measurements (Figure 2) clearly reveal the columnar prior β grains thanks to the correlation between the martensite needles’ orientations and the β phase one [18]. These grains grow across several layers thanks to the epitaxial growth mechanism. The images of the AB samples show that martensite needles can be seen in both cases. Ti6246 needles contain some features that could be associated with both the β → α’’ transformation and the accommodation of stresses generated during the building process [19]. Both micrographs reveal the presence of hierarchical structures characterized by primary and secondary martensite needles (Figure 3a,b). These features are often found in titanium alloy microstructures processed by AM methods as they are caused by the unique thermal history to which the material is subjected [7].

The microstructural evolution that arises during the post-processing heat treatments can be understood by comparing the images of the AB and heat-treated (HT) Ti64 and T6246 samples reported in Figure 3. In both cases, the martensite decomposes to α + β. As the heat treatments selected were below the β-transus temperature, columnar prior β grains are still visible.

**Figure 4:**
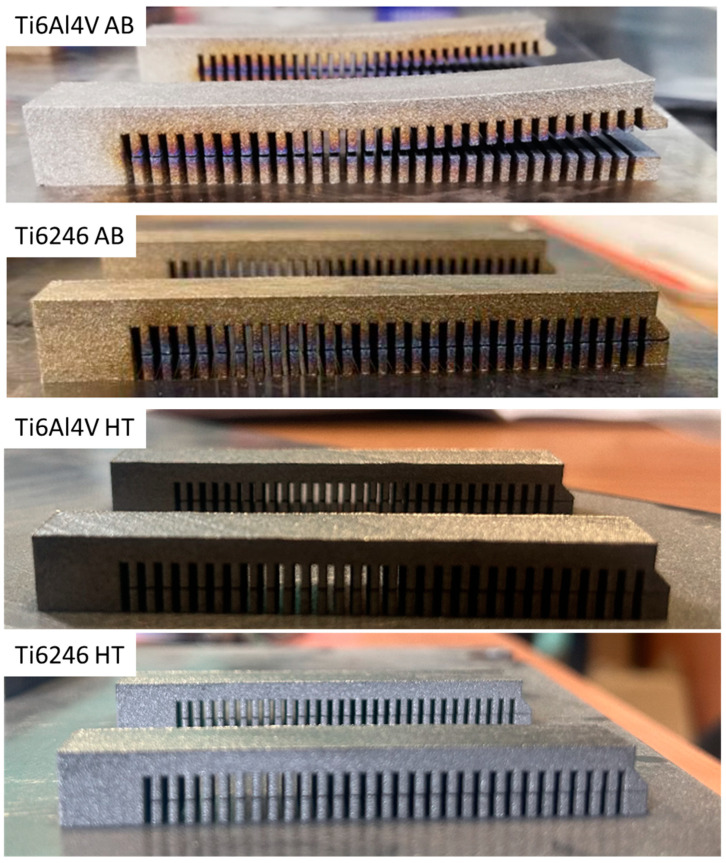
Images of as-built and heat-treated Ti6Al4V and Ti6246 cantilevers after WEDM cut.

Images of the Ti6Al4V and Ti6246 cantilevers and the post-WEDM cut and deflection values are reported in Figure 4 and Table 4, respectively. It is clear that AB Ti6246 showed only minimal distortion (about 0.2 mm), while all AB Ti6Al4V cantilevers showed high deflection values of about 2.9 mm. The orientation of the cantilever on the build plate does not affect the distortion values, probably thanks to the 67°-rotated stripe scanning strategy used [20]. Similar results for Ti6Al4V were reported by Siewert et al. and Cardon et al., who used similar energy density values [20,21]. Higher deflection values were instead reported by Pauzon et al. [22], probably due to the different building parameters used. The large distortion values of Ti6Al4V could be due to its high sensitivity to the thermal gradient generated along the layers related to its thermophysical properties (i.e., high yield strength, high Young’s modulus, and low thermal conductivity) [23]. In Ti6246, on the contrary, the low yield strength of the material due to the soft α’’ martensite allows for plastic deformation; the material could therefore be able to accommodate the stresses generated by the strong thermal gradient. Evidence of this deformation can be found in the twins found in Figure 2 and Figure 3.

It is interesting to note that the post-processing heat treatment strongly reduces the warping of Ti6Al4V, while it only has a minor effect on the maximum distortion of Ti6246 cantilevers.

**Figure 5:**
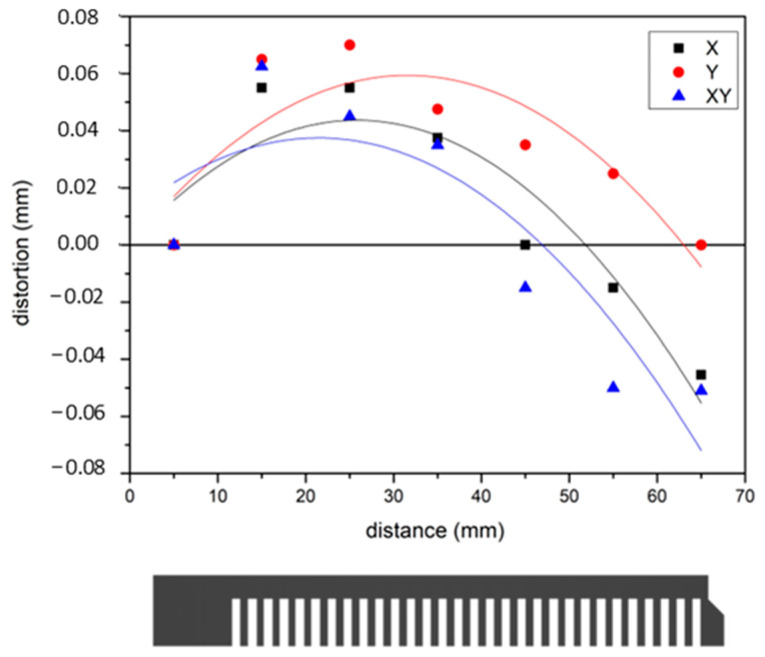
Detailed measurement of heat-treated Ti6246 cantilevers built along different orientations.

To further investigate the effect of the post-processing heat treatment on Ti6246, the geometries of the heat-treated Ti6246 cantilevers were measured in more detail along their length (see Figure 5). It can be noticed that, after the heat treatment, the cantilever was concave, as opposed to the convex shape of as-built cantilevers.

**Figure 6:**
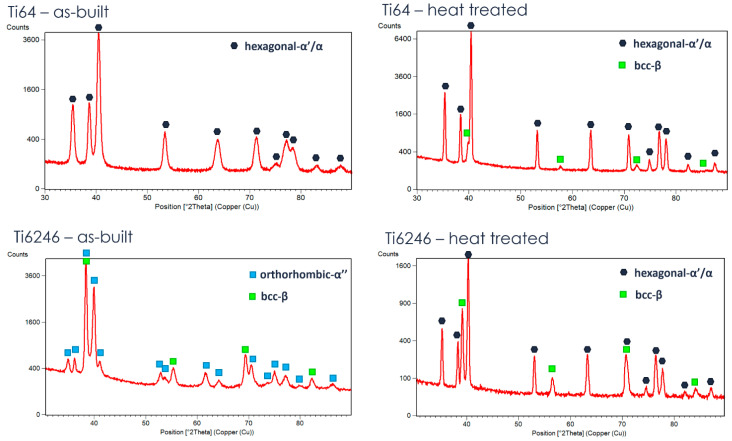
XRD diffractogram of Ti6Al4V and Ti6246 in the as-built and heat-treated conditions.

The XRD measurements (Figure 6) confirm the considerations made about the microstructure: the AB Ti6Al4V is mainly constituted by α’, while AB Ti6246 is made of α’’. In both cases, the martensite decomposes to α+β during the heat treatments, with a higher amount of β in Ti6246. The von Mises equivalent residual stress measurements reported in Table 5 are in line with the cantilever measurements reported in Table 4. The comparison of the values indicates that Ti6Al4V as-built samples have the highest stress values (494 MPa), which, however, are strongly reduced due to the stress-relieving heat treatment (44 MPa). The residual stress behavior of Ti6246 is completely different, showing only minor stresses in the as-built state (191 MPa). The low stresses in the as-built state could be due to the presence of the soft orthorhombic martensite that accommodates the deformations due to the material’s thermal history, as confirmed by the presence of twins in Figure 2. It is interesting to underline that these stresses were not reduced by the post-processing heat treatment. This is surprising because, as demonstrated by Carrozza et al. [8] by Differential Scanning Calorimetry, a temperature of 750 °C is sufficient for the residual stress relaxation of Ti6246. Therefore, we suggest that this residual stress of the heat-treated samples could be due to the volume changes related to the α″ → α + β transformation (Table 6). This hypothesis is in line with the opposite deflection of the cantilever shown in Figure 5. It is, however, surprising that this does not happen for Ti6Al4V during the α′ → α + β transformation. To investigate this effect, the main properties involved in residual stress generation and relaxation are reported in Table 6.

The comparison of the data indicates that Young’s modulus, the Coefficient of Thermal Expansion, and the thermal conductivity of the two alloys are quite similar, suggesting that the stresses generated due to the strong thermal gradient in the LPBF process should be similar (Table 6). However, the low yield strength value of AB Ti6246 allows for the partial accommodation of those stresses. The β-transus temperatures reported in Table 6 confirm that none of the heat treatments cause the martensite to undergo β transformation but that in both cases an α′/α″ → α + β transformation arises. The phenomena arising during the Ti6Al4V heat treatment were deeply investigated by Kaschel et al. [24], who proposed a mechanism of stress relaxation as follows: from 25 to 400 °C, the lattice parameters of the compressed α′ lattice slightly increase thanks to the diffusion of Al and V. At temperatures higher than 400 °C, V diffuses out of the lattice leading to the α′ → α + β decomposition. This transformation is facilitated by temperatures higher than 700 °C thanks to the expansion of the lattice that facilitates the Al and V diffusion. To date, a similar study on Ti6246 is not available in the literature; however, the lattice parameter evolution during this α″ → α + β transformation in Ti6246 was studied by Otte et al., who observed that this transformation is associated with lattice strains [25]. Therefore, we suppose that this could be the reason for the relatively high stress measured on the HT Ti6246 specimens.

**Table 6:** Ti6Al4V and Ti6246 properties [9,26,27].

	Ti6Al4V AB	Ti6Al4V HT	Ti6246 AB	Ti6246 HT
Young’s modulus RT (GPa)	100–110		103	
Yield strength (MPa)	800–1280	950–960	483 ± 6	1052 ± 20
Coefficient of Thermal Expansion (10^−6^/K)	8.60	-	8.60	-
Thermal conductivity (W/(m.K))	6.7	-	7.7	-
Beta-transus temperature (°C)	800		880	

## 4. Conclusions

In the present work, the residual stresses of Ti6Al4V and Ti6246 alloys processed by Laser Powder Bed Fusion (LPBF) were evaluated via the cantilever method and via the sin^2^(Ψ) X-ray diffraction (XRD) method. In all cases, the cantilever results demonstrated that, thanks to the 67°-rotated scanning strategy, these stresses are not related to the samples’ orientations. The comparison of the alloys indicated that Ti6Al4V had the highest residual stresses, which are due to the shrinkage during the cool-down phase due to the steep thermal gradient, high Young’s modulus, and yield strength of the α’ martensite. The post-processing stress-relieving heat treatment performed on the samples at 800 °C for 2 h was able to strongly reduce these stresses, as demonstrated by both the cantilever distortion and the XRD measurements. The Ti6246 alloy showed the opposite behavior, showcasing relatively low stresses in the as-built state. These residual stresses could be due to the presence of the soft α’’ martensite that accommodates the deformation due to the building process. It is interesting to note that these residual stresses were not reduced by the post-processing heat treatment, probably due to the volume change related to the α’’martensite to α + β transformation.

These results could be very interesting from an industrial point of view as using Ti6246 instead of Ti6Al4V could facilitate the LPBF production of extremely complex parts. Evidently, this material change must also take into account two other aspects: first, the requirements dictated by the application must be reached, then the cost impact should also be considered.

## Figures and Tables

**Table 1:** Chemical compositions of the powders, as measured by the suppliers.

wt.%	Ti	N	C	H	Fe	O	Al	V	Mo	Zr	Sn	Other
Ti6Al4V	Bal.	0.003	0.01	0.002	0.183	0.083	5.86	3.99	-	-	-	-
Ti6246	Bal.	0.005	0.01	0.002	0.031	0.118	5.96	-	5.89	3.67	1.85	<0.3

**Table 2:** Building parameters used for Ti6Al4V and Ti6246.

	Ti6Al4V	Ti6246
Laser power (W)	170	190
Scanning speed (mm/s)	1250	1100
Hatching distance (mm)	0.1	0.1
Layer thickness (μm)	30	30
VED (J/mm^3^)	45.3	57.6
Platform temperature (°C)	100	100
Scanning strategy	67° rotated	67° rotated

**Table 3:** Heat treatment procedures for Ti6Al4V and Ti6246.

	Temperature and Time	Pressure	Cooling
Ti6Al4V	800 °C for 2 h	10^−4^ mbar	Slow cooling of approximately 1.5–2.0 °C/min
Ti6246	750 °C for 2 h	10^−4^ mbar	Slow cooling of approximately 1.5–2.0 °C/min

**Table 4:** Cantilever distortion results.

	Z As-Built (mm)	Max Z After EDM Cut (mm)	Max Distortion (mm)	Z As-Built (mm)	Max Z After EDM Cut (mm)	Max Distortion (mm)
	Ti6Al4V as-built			Ti6246 as-built		
Y	9.05	11.91	2.86	8.97	9.17	0.20
X	8.98	11.91	2.93	9.12	9.30	0.18
45°	9.01	11.89	2.88	9.12	9.41	0.29
	Ti6Al4V HT			Ti6246 HT		
Y	9.07	9.14	0.07	9.15	9.20	0.05
X	8.99	9.03	0.04	9.15	9.24	0.09
45°	9.11	9.18	0.07	9.15	9.21	0.06

**Table 5:** Residual stress measured with the sin^2^(Ψ) method expressed as von Mises equivalent stress.

	Ti6Al4V	Ti6246
AB	494 ± 31 MPa [α′/α—(211)]	191 ± 17 MPa [α″—(221)]
HT	44 ± 3 MPa [α′/α—(211)]	203 ± 10 MPa [α′/α—(211)]

## Data Availability

Data are contained within the article.
